# The Story of New York Briefly Told

**Published:** 1899-12

**Authors:** 


### THE STORY OF NEW YORK BRIEFLY TOLD.

(Continued from page 729, November number.)

One of the most attractive and instructive features of the Convention in New York was the extraordinary and remarkable collection of pathological specimens furnished by the representatives of the profession engaged in inspection work in the Bureau of Animal Industry.

Through the courtesy of Eastman & Co., of Fifty-ninth Street and Eleventh Avenue, and the kindly assistance of their corps of employés, these fresh specimens were exhibited on improvised tables, where every one had ample opportunity of inspection and the value of a practical lesson of the importance of this branch of food inspection.

Some of the specimens were unique in pathological appearance, and while given local names were not among the list of recorded studied changes in pathology, they awakened a deal of interest among the unusually large number who availed themselves of this special privilege. The Association is greatly indebted to the Bureau inspectors for their labors, and the several veterinary colleges exhibited a keen interest in obtaining for their museums a goodly share of these valuable specimens:

| | | | |
|---|---|---|---|
| 1. | Steer, | Tuberculosis. | Heart. |
| 2. | Cow, | " | Lungs and part of thorax. |
| 3. | Sow, | " | Lungs and heart. |
| 4. | Cow, | " | Tibia. |
| 5. | Hog, | Tubercular arthritis. | |
| 6. | " | Tuberculosis. | Lung and liver. |
| 7. | " | " | Lung, liver, and intestines. |
| 8. | Cow, | " | Mammary gland. |
| 9. | Sheep, | Nodular disease. | Intestines. |
| 10. | " | Jaundice. | |
| 11. | Hog, | " | |
| 12. | " | " | |
| 13. | " | Chronic cholera. | |
| 14. | " | Cholera. | |
| 15. | Steer, | Actinomycosis. | Inferior maxilla. |
| 16. | " | " | Superior " |
| 17. | " | " | " " |
| 18. | " | " | Post-pharyngeal gland. |
| 19. | " | Chronic Glossitis. | |
| 20. | Calf, | Texas fever. | |
| 21. | Sheep, | Ictero-hæmaturia. | |
| 22. | Steer, | M. M. œsophagus. | Parasites. |
| 23. | Cow, | Mammitis. | |
| 24. | Hog, | Tuberculosis. | Bone. |
| 25. | " | Cysticercus cellulosus. | Tongue and part of psoas. |
| 26. | Steer, | Hydrocele. | |
| 27. | Sow, | Purulent nephritis. | |
| 28. | Steer, | Abnormal foot. | |
| 29. | Hog, | Abscess of kidney. | |
| 30. | Sow, | Extra-uterine pregnancy. | |
| 31. | " | " " " | |
| 32. | " | " " " | |
| 33. | " | Tuberculosis. | Cervical vertebra. |
| 34. | " | Fœtus, 14 weeks. | |
| 35. | Sow, | Uterus, 14 weeks pregnant. | |
| 36. | Hog, | Tumor of kidney. | |
| 37. | Steer, | Actinomycosis. | Lungs. |
| 38. | Hog, | Cysticercus cellulosus. | Heart. |
| 39. | Sow, | Echinococcus vet. | |
| 40. | Hog, | Cystic liver. | |

| | | | |
|---|---|---|---|
| 41. | Steer, | Actinomycosis. | Lungs. |
| 42. | Sow, | Ovarian cysts. | |
| 43. | " | Ovary nearing œstrum. | |
| 44. | Hog, | Liver abnormal in shape. | |
| 45. | Cow, | Tuberculosis. | Sternum. |
| 46. | Hog, | " | Heart. |
| 47. | " | Chronic hepatitis. | |
| 48. | Steer, | Reticulum. | Foreign bodies. |
| 49. | Hog, | Skin disease. | |
| 50. | " | Diamond skin disease. | |
| 51. | " | Tuberculosis. | Hock. |
| 52. | Sow, | " | Part of thoracic wall. |
| 53. | Steer, | Pleurisy. | |
| 54. | Hog, | Tuberculosis. | Pleura. |
| 55. | " | " | " |
| 56. | " | Skin disease, 2 specimens. | |
| 57. | " | Tuberculosis. | Vertebra. |
| 58. | " | Ringworm. | |
| 59. | Cow, | Tuberculosis. | Bone. |
| 60. | Hog, | Cholera. | Viscera. |
| 61. | ? | ? | ? |
| 62. | Sheep, | Cysticercus tenuicollis. | |
| 63. | Sow, | Tuberculosis. | Liver and lymphatics. |
| 64. | Steer, | Nephritis. | |
| 65. | Hog, | Degenerated kidney. | |
| 66 | Steer, | Liver. | Multilocular cysts. |
| 67. | " | Cystitis. | |
| 68. | " | M. M. œsophagus. | Parasites. |
| 69. | Cow, | Eye. | Contusion. |
| 70. | Hog, | Testicle. | Adenoma. |
| 71. | " | Cystic kidney. | |
| 72. | " | " " | |
| 73. | " | " " | |
| 74. | " | Kidney abscess. | |
| 75. | Sow, | " " | |
| 76. | Sheep, | Neoplasm. | |
| 77. | Hog, | Cystic kidney. | |
| 78. | " | Large white kidney. | |
| 79. | " | Adenoma of kidney. | |
| 80. | " | Cystic kidney. | |
| 81. | " | Kidney. | ? |
| 82. | Steer, | Multiple abscess. | Liver. |
| 83. | " | Hepatic abscess. | |
| 84. | " | " " | |

---

Collar-buttons made from milk is one of the latest uses of this lacteal fluid. If they won't break, chafe the neck, or fall out of one's shirt in a sleeping-car berth, men will rise up with one accord and say, "God bless the cow!"

